# ADVANCING OUR UNDERSTANDING OF ELDERHOOD: A NARRATIVE REVIEW

**DOI:** 10.1093/geroni/igz038.2562

**Published:** 2019-11-08

**Authors:** Jenny Inker, Tracey Gendron, Marshall Brooks

**Affiliations:** 1 Virginia Commonwealth University, Richmond, Virginia, United States; 2 Virginia Commonwealth, Richmond, Virginia, United States

## Abstract

There are relatively few explorations of later life in the peer reviewed gerontological literature that holistically embrace the duality of potential and decline. This is in striking contrast to a growing body of non-scholarly literature, frequently authored by elders, displaying deep interest in the phenomenon of elderhood, i.e. the holistic, lived experience of later life. We conducted a narrative review with the aim of describing the state of the science with regard to the bio-psycho-social-spiritual experience of elderhood. Following a search of multiple databases for English language, peer reviewed articles published from 2000-2017, we identified 24 articles in the disciplines of gerontology, anthropology, psychology, the humanities, and spirituality studies, reflecting elderhood in Eastern and Western cultures. While the articles offered no shared operational definition of elderhood, nor applied any unifying conceptual or theoretical structures, several common themes emerged. These included the description of elderhood as both inward facing (inner development) and outward facing (social contributions of elders). Numerous articles also recognized that ageism socially mediates the experience of elderhood, resulting in a failure of social systems and structures to recognize or provide opportunities for lifelong growth in later years, including a lack of mentors and role models for individuals transitioning into elderhood. This review demonstrates that there is a compelling need for the discipline of gerontology to strengthen our understanding of the phenomenon of elderhood by leading on the development and implementation of theoretically driven empirical research into the subject of the holistic, lived experience of later life.

